# Evaluation of a Silicon Carbide Static Induction Transistor for High Frequency/High Temperature Sensor Interface Circuits: Measurements and Modeling

**DOI:** 10.3390/s25227051

**Published:** 2025-11-18

**Authors:** Jonathon R. Grgat, Maximilian C. Scardelletti, Christian A. Zorman

**Affiliations:** 1 Department of Electrical, Computer, and Systems Engineering, Case Western Reserve University, Cleveland, OH 44106, USA; jonathon.grgat2@gmail.com; 2 Communications and Intelligent Design Division at NASA Glenn Research Center, Cleveland, OH 44135, USA; maximilian.c.scardelletti@nasa.gov

**Keywords:** silicon carbide, static induction transistor, scattering parameters, transconductance, gain, maximum stable gain, small signal model

## Abstract

**Highlights:**

**What are the main findings?**

- Characterization of a SiC SIT at high temperatures and high frequencies;
- Development of a small signal model for high-temperature/high-frequency operation.

**What is the implication of the main finding?**

- Demonstrates potential of the SiC SIT for wireless sensor interfaces.

**Abstract:**

In this paper, we report on the characterization of a silicon carbide static induction transistor (SiC SIT) for potential use in sensor interface circuits that operate at frequencies up to 100 MHz and temperatures up to 400 °C. Measurements were performed to generate current–voltage curves, capacitive transistor characteristics, and high-frequency scattering parameters at temperatures between 25 and 400 °C. The measured data were used to extrapolate the transconductance, g_m_, as a function of temperature and to develop a small signal model. Circuit simulation tools were used to generate scattering parameters, which were compared to the measured values. At 400 °C, the maximum difference between the measured and simulated scattering parameters for frequencies from 20 to 100 MHz were all less than 0.1 dB, indicating strong agreement between the model and measurement results. The average transition frequency, f_t_, calculated from measured parameters was 197.8 MHz, which compares favorably to the simulated value from the model (200 MHz). This is also the first paper to report the characterization of a SiC SIT at temperatures above 100 °C. The high-temperature model is the first of its kind for a silicon carbide static induction transistor and the findings reported herein provide a platform to stimulate further development for sensor interface circuits that require transistors that operate at both high frequency and high temperature.

## 1. Introduction

Next-generation gas turbine engine health monitoring for high performance jet engine-powered aircraft incorporates integrated microelectronic sensors specifically designed to enhance aircraft functionality, engine efficiency, and safety. Temperature, air flow, and pressure associated with the gas path as well as emissions from the engine correlate to the state of the combustor and other engine health conditions [1]. Temperature sensing and flow measurements are critical performance parameters to assess the fuel consumption efficiency and total gas velocity [2,3]. The ability to measure variable changes in emissions, temperature, blade tip clearance, and pressure are also desired to improve engine performance [4,5,6,7,8,9,10]. Monitoring these parameters accurately requires positioning electronic sensors in the hot zone of the gas turbine engine, which exposes them to temperatures in excess of 400 °C. At these temperatures, small signal amplification is needed at the transducer level to distinguish the desired measured signal from the significant electronic noise generated by the harsh environment. Moreover, the most demanding applications are associated with locations that can only be practically accessed by wireless connections, necessitating the need for on-board amplifiers, oscillators, mixers, and other radio frequency (RF) electronic circuits that exhibit stable operation at high temperatures. Because antenna size is inversely proportional to frequency, electronics that operate at high frequencies are needed to meet the demanding size and form factor requirements of these sensor systems. The need for this technology is not limited to aerospace applications, in fact, other applications that will benefit from the development of high-temperature RF electronic circuits include sensor systems for deep-well drilling for oil and gas extraction [11,12,13] as well as exhaust monitoring sensors for automotive systems [14], to name a few.

Silicon carbide (SiC) and gallium nitride (GaN) are two of the leading semiconductors for high-temperature electronics due to their wide bandgap. Examples of GaN-based devices include an AlGaN/GaN-on-Si high electron mobility transistor (HEMT) that exhibited stable operation to 500 °C under DC conditions [15] and a GaN-based RF power amplifier with a center operational frequency of 97.5 MHz and gain of 27 dB at temperatures up to 230 °C [16]. SiC, is, however, the leading semiconductor for high temperature integrated sensor applications due to its outstanding physical properties, wafer-scale microfabrication techniques, mature transistor technologies, and rapid advances in the development of physical and chemical sensors [17,18,19]. High-temperature operation of SiC-based transistors have been demonstrated, stimulating the development of SiC-based amplifier circuits for high-temperature applications. Successful examples include a SiC operational amplifier with a peak gain of 39.46 dB and a unity-gain at 4.36 MHz operating at 500 °C [20], a bipolar junction transistor (BJT)-based high-temperature amplifier with a maximum operational temperature of 251 °C at 54.6 MHz [21], and a junction field effect transistor (JFET)-based differential amplifier with an operational gain of 50.8 dB and a gain unity of approximately 8.5 MHz at 500 °C [22].

Unfortunately, electronic circuitry of the type needed for the high-temperature wireless sensor applications described previously require transistors that exhibit stable operating frequencies considerably above the state-of-the-art (~5 MHz) to meet the desired performance and size requirements. To the best of our knowledge, no such amplifier has been demonstrated in SiC. To address this gap, this paper explored the potential of using the SiC static induction transistor (SIT), a device developed specifically for high-frequency/high-power applications, as an active device in low power wireless microsystems by characterizing and modeling its high-frequency performance at high temperatures by focusing on low power operation at 400 °C and frequencies between 20 and 100 MHz.

**Figure 1:**
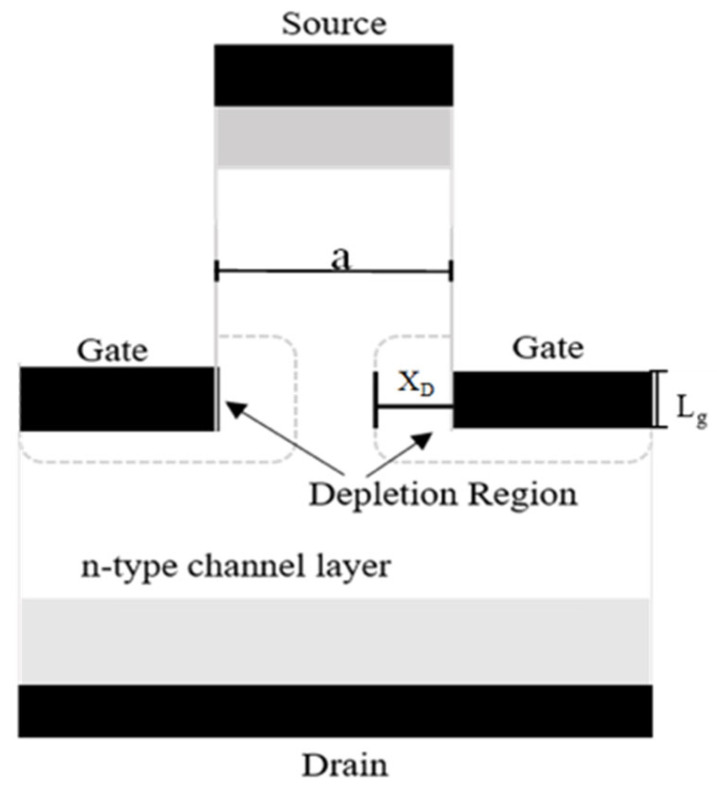
Cross-sectional schematic diagram of a conventional SIT indicating the depletion regions associated with pinch-off.

Figure 1 depicts the basic structure and design of a typical SIT. This normally on device operates via negative gate modulation with Schottky contacts to control the flow of current when a positive bias is applied to the drain. The device itself operates in three regimes, but unlike metal semiconductor field effect transistors (MESFETs) and JFETs, it does not saturate like these FET-based transistors. This is due to a smaller source/gate length ratio. Instead, for a large drain voltage and a small negative gate bias, the device operates in the space-charge limited flow regime and the current increases with an increasing drain voltage. This region allows for a larger linear gain and lower biasing to limit self-heating. The SIT is comparable to a vertical-channel field effect transistor (v-FET) in which the distance between the source and depletion layer of the drain is small enough that the negative feedback of the channel resistance does not affect its direct current characteristics.

The SIT was originally developed in silicon for applications that included induction heating, high-power, high-voltage power supplies as well as ultrasonic generation [23,24,25]. The first SiC SIT devices capitalized on the high thermal conductivity, increased breakdown voltage, and high saturation velocity of SiC relative to Si [26]. Currently, SiC SITs are used in commercial high-power and high-frequency applications including radar and transmitter circuitry [27]. It has been suggested that SiC SITs could operate at temperatures as high as 600 °C due to the high-temperature properties of SiC [28,29]. The robust current control offered by the unique SIT geometry suggests that the SiC SIT could be an excellent candidate for high-temperature applications that require high operating frequencies. To the best of our knowledge, this paper describes the first effort to characterize and develop a model for a SiC SIT operating at temperatures up to 400 °C specifically for the RF range.

## 2. High Temperature Characterization

### 2.1. Overview

**Figure 2:**
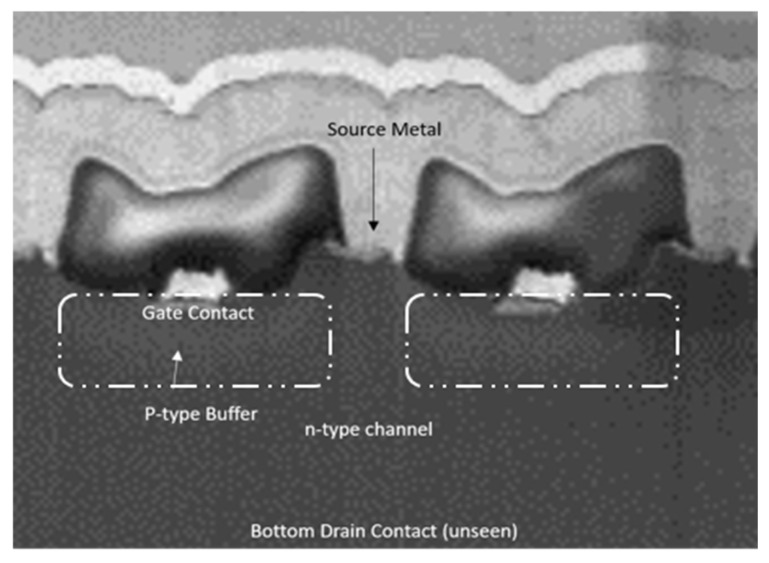
Cross-sectional FIB-SEM image showing the key functional regions of a SiC SIT.

The SiC SIT characterized in this paper was a commercially available device acquired from Microsemi^TM^ (Chandler, AZ, USA) and utilized the dual mesa technology [30]. The Microsemi^TM^ SiC SIT functions similarly to that of the device illustrated in Figure 1 but with one difference. Instead of using metal-based Schottky gate contacts to induce depletion regions in the n-type channel, acceptor dopant implantations under the gates are used to create p–n junctions, thus enabling higher blocking voltages than a metal gate Schottky contact-based SIT. A cross-sectional scanning electron microscope (SEM) image of a representative device is presented in Figure 2. Here, one can see the acceptor implanted gate junctions that extend the gate perimeter toward the source (labeled “p-type Buffer”). The SiC SIT used in this paper was specifically designed for high power applications, but continuous low power operation could yield promising results under high temperature conditions due to the small bias required to achieve pinch-off in this device geometry. Other SiC SITs with Schottky contacts have been investigated up to 100 °C, but this is the first report regarding high temperature characterization for an acceptor-implanted SiC SIT while extending the temperature range for SiC SITs in general from 100 °C to 400 °C for RF applications [27].

### 2.2. DC Measurements

High temperature current–voltage (I–V) and capacitive–voltage (C–V) measurements were performed using a custom-built high temperature probe station. High temperature chip carriers consisting of 10 μm-thick Au, 150 µm-pitch, ground-signal-ground (GSG) probe pads on a 500 µm CoorsTek 996 Alumina Superstrate, Golden, CO, USA were used to facilitate the electrical contact between the SIT and measurement system. High-temperature silver epoxy was used to adhere and provide a conductive connection between the device and the metalized substrates. Gold wire bonds were used for electrical connection between the device drain, gate, and source contact pads as well as substrate contact pads. High-temperature Au/W needle probes were used to make electrical connection from the SIT devices to the characterization equipment via co-axial cables. The devices were heated using a custom-built, high-temperature chuck comprised of a ceramic heater and a space shuttle tile. The heater was powered by a Keysight^TM^ N5769A power supply, Santa Rosa, CA, USA and the temperature controlled by a LabVIEW^TM^ PID controller. I–V curves were recorded at 25, 100, 200, 300, and 400 °C utilizing a Keysight^TM^ B1505A Power Device Analyzer, Santa Rosa, CA, USA. Measurements were taken after the device reached a stable temperature for each increment, which was within 1% of the desired temperature.

**Figure 3:**
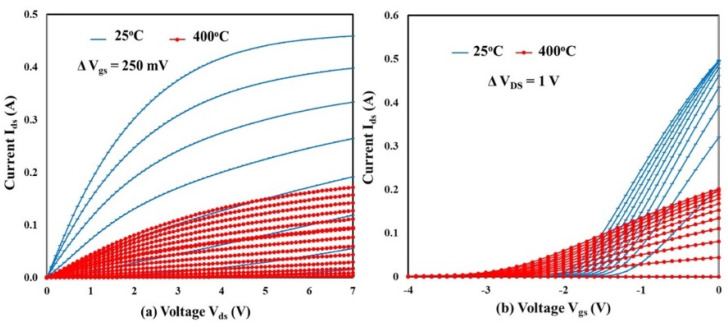
I−V characteristics of a SiC SIT at 25°C and 400°C: (**a**) I_ds_ versus V_ds_ and (**b**) I_ds_ versus V_gs_.

Representative I–V characteristics for the SiC SIT at 25 and 400 °C are presented in Figure 3. The I_ds_–V_ds_ measurements were performed by sweeping V_ds_ from 0 to 7 V while increasing the gate bias incrementally by 250 mV from −4 to 0 V for each sweep (Figure 3a). The observed decrease in drain current at 400 °C is related to a decrease in electron mobility due to the effect of temperature on the saturation velocity of the majority carriers [27]. I_ds_–V_gs_ characteristics are shown in Figure 3b, where V_gs_ was swept from −4 V to 0 V while increasing V_ds_ from 0 V to 10 V over increments of 1 V. Figure 3b shows the strong influence of temperature on the gate bias required to achieve pinch-off of the channel. The increase in temperature elicits thermionic conduction, shifting the required pinch-off voltage from −1.9 V_gs_ at 25 °C to −3.5 V_gs_ at 400 °C.

**Figure 4:**
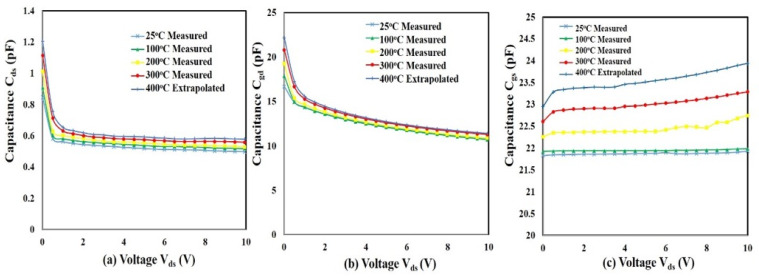
C–V measurements of a SiC SIT at temperatures between 25 °C and 400 °C using a gate bias of −3 V_gs_: (**a**) C_ds_ vs. V_ds_. (**b**) C_gd_ vs. V_ds_ and (**c**) C_gs_ vs. V_ds_. For each capacitance measurement, V_ds_ was swept from 0 to 10 V.

C–V measurements were performed utilizing the Keysight^TM^ B1505A Power Device Analyzer at temperatures of 25, 100, 200, and 300 °C as V_ds_ was swept from 0 V to 10 V and the gate voltage was held constant at −3 V. The three junction capacitances of the SiC SIT, specifically the drain-source capacitance, C_ds_, the gate-drain capacitance, C_gd_, and the gate-source capacitance, C_gs_ are shown in Figure 4. In this instance, each terminal was measured when the device was operating in a non-conducting mode, since the vertical SIT is a normally on device. This was achieved by applying a negative bias on the gate to ensure an approximate zero drain current. The analyzer was calibrated using an open/short calibration with the Au/W probes. Capacitor calibration standards of known values were then measured to ensure accuracy of the measurement system. C_ds_ and C_gd_ remained constant with increasing temperature while a small increase was observed for C_gs_ at temperatures up to 300 °C. At 400 °C, it was observed that the drain began to experience low-level conduction. Due to limitations associated with the ability of the B1505A analyzer to handle micro-amp currents, C–V measurements could not be performed at 400 °C. Instead, the capacitance data at 400 °C were determined by extrapolating the capacitance at each voltage from the measured capacitance at lower temperatures.

### 2.3. Scattering Parameter Measurements

**Figure 5:**
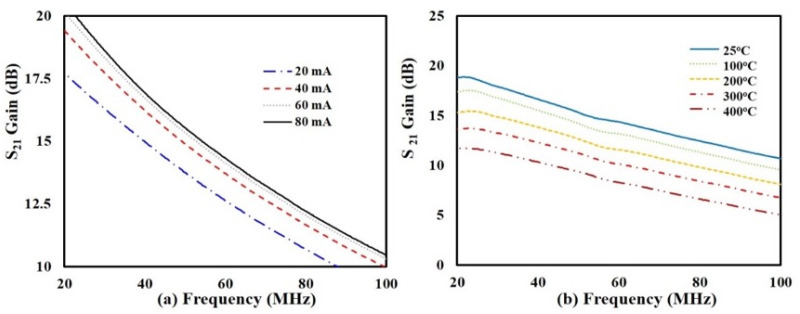
S_21_ gain as a function of frequency for a SiC SIT: (**a**) at 25 °C for drain currents between 20 and 80 mA, and (**b**) at a drain current of 40 mA and temperatures ranging between 25 and 400 °C.

Scattering parameters (S-parameter) were measured by an Agilent^TM^ E836B Network Analyzer (Santa Clara, CA, USA) using 150 μm-pitch, high temperature probes (GGB Industries^TM^ Model P-12-9403), Naples, FL, USA. Two bias-tees were placed in series at port-1 and port-2 between the analyzer and RF cables, and two external power supplies were used to bias the drain and gate of the transistor while keeping the source grounded. Network analyzer calibration was performed using a short-open-load-thru (SOLT) calibration substrate, which established a reference plane at the probe tips. Measurements were made from 20 to 100 MHz at temperatures of 25, 100, 200, 300, and 400 °C. The unmatched transistor gain, S_21_, at 25 °C is shown in Figure 5a for drain currents, I_ds_, of 20, 40, 60, and 80 mA. The plot indicates that as the drain current increased from 20 to 80 mA, S_21_ increased over the entire range of frequencies. The measured data demonstrate that S_21_ is dependent on the drain current, which was expected. An I_ds_ of 40 mA was chosen as the operational drain current for the SiC SIT primarily because its S_21_ gain at 50 MHz (14.9 dB) lay in the mid-range of the distribution. An I_ds_ of 40 mA provides a sufficiently high gain at room temperature to enable tuning/adjustment in biasing when operated at 400 °C while simultaneously keeping transistor operation in low power mode. As stated previously, although the SiC SIT was developed specifically for high power applications, it has potential for high-temperature applications provided that it is operated under low power conditions, and therefore the trade-off between S_21_ and I_ds_ is an important consideration.

Figure 5b plots S_21_ for an I_ds_ of 40 mA at temperatures from 25 to 400 °C. Figure 5b shows an unmatched transistor gain loss at 50 MHz of 5.9 dB as the temperature increased from 25 °C to 400 °C, a 38% decrease over this temperature range. More importantly, the unmatched transistor gain at 400 °C was 9.35 dB at 50 MHz, which is sufficient for a wide range of wireless sensor applications. This loss in gain can be attributed to temperature-dependent changes in the transconductance, carrier mobility, and internal resistances in the transistor.

The transconductance, g_m_, was calculated from the measured I_ds_–V_gs_ curves at 25, 100, 200, 300, and 400 °C using Equation (1):

(1) $${\left.\frac{\Delta Ids}{\Delta Vgs}=g_{m}\right|}_{Vds=constant}$$

**Figure 6:**
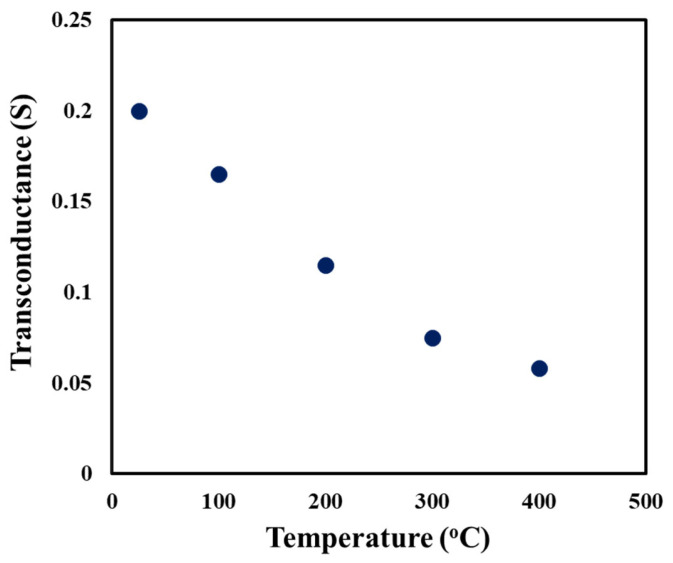
Transconductance of the SiC SIT as a function of temperature for a drain current of 40 mA.

For each calculation, V_gs_ was decreased to maintain an I_ds_ of 40 mA while the drain voltage, V_ds_, was held constant at 7 V. Figure 6 plots the transconductance as a function of temperature. The measurement uncertainty in g_m_ was 1 part in 1000. The transconductance was observed to decrease from 200 mS at 25 °C to 60 mS at 400 °C.

**Figure 7:**
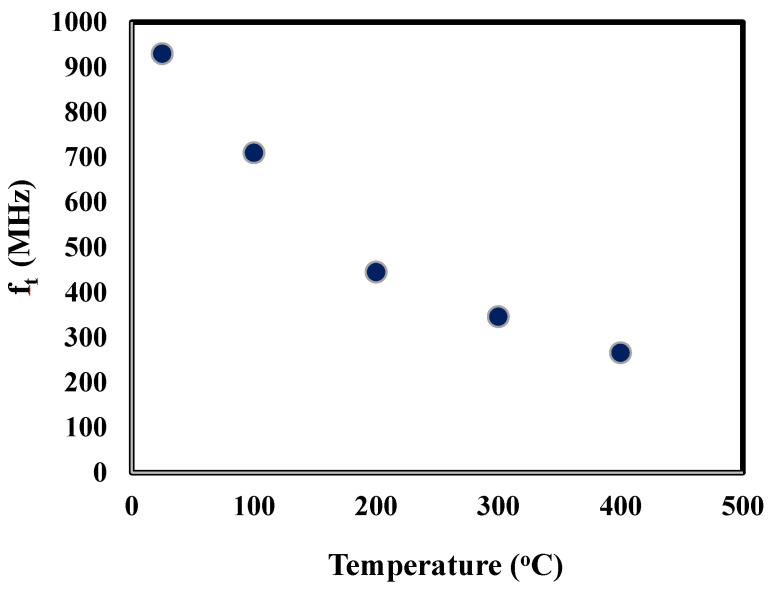
Calculated transition frequency, *f*_t_, for temperatures between 25 and 400 °C.

The temperature-dependent behavior of the transition frequency, also known as the cut-off frequency, *f_t_*, was calculated using Equation (2):

(2) $$f_{t}=\frac{g_{m}}{2\pi (C_{gs}+C_{gd})}$$

where g_m_ was determined from Figure 3b and C_gs_ and C_gd_ from Figure 4. Figure 7 is a plot of the transition frequency, *f_t_*, with respect to temperature. The measurement uncertainty in *f_t_* was ~5%. At 400 °C, the *f_t_* was 270 MHz, which was 5.4 times greater than the target operating frequency of 50 MHz, indicating that the SiC SIT is a viable option for an amplifier, oscillator, and other active circuity operating in a 50 MHz range at 400 °C, outperforming other previously reported active devices in this regard [20,22].

## 3. SiC SIT Modeling

**Figure 8:**
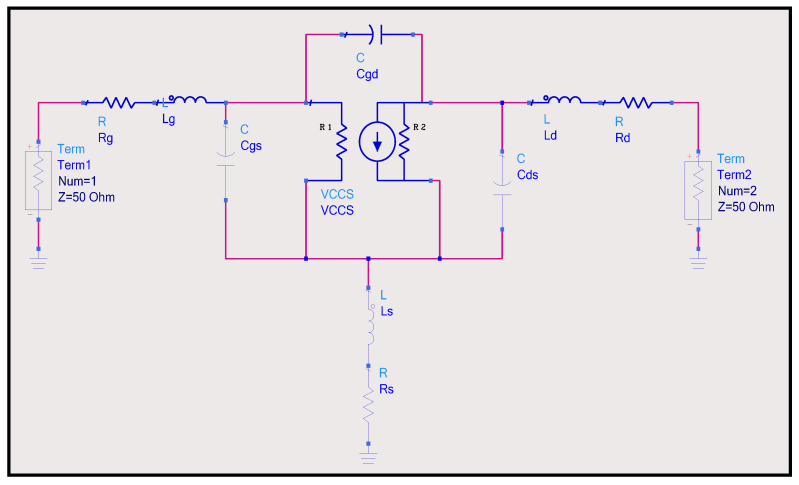
Schematic circuit diagram of the small signal model for the SiC SIT with the parasitic resistance/inductance components incorporated to accurately model the behavior at elevated temperatures.

Because key features of the SiC SIT are difficult to accurately quantify, a modeling approach based on a small signal model was chosen to simulate the electrical behavior of the transistor. Using the transconductance values from Figure 7 and the C–V characteristics from the measured devices in Figure 4, a model was developed in Keysight’s Advanced Design Systems (ADS) [31,32]. We used the measured C–V intrinsic values and resulting transconductance in the model to determine other parasitic values that are difficult to measure. Figure 8 represents the circuit schematic for the SiC SIT small signal model. This conventional model consists of a voltage controlled current source (VCCS) which represents the transconductance of the device, R1 and R2, which are the internal resistances associated with the VCCS, the junction capacitances C_ds_, C_gd_, and C_gs_, and terminal parasitic inductive/resistive components, L_s_/R_s_, L_d_/R_d_, and L_g_/R_g_. Fifty Ω terminals/ports were used in ADS to match the 50 Ω reference impedance of the Agilent^TM^ E836B Network Analyzer. The Circuit Optimization engine in ADS was used to determine unknown inductive and resistive parasitic values to refine the model. The model could then be used to accurately represent the SiC SIT for the design of amplifiers, oscillators, and other circuits that require this SiC SIT as the active device. Optimization is an automated procedure of achieving the desired circuit performance by tuning unknown component values needed to meet the specific optimization goals. To ensure component values were within an acceptable range, practical components limits were preselected. Unknown values were determined via algorithms to fit the new simulated model performance against the measured S-parameters of the SIT. The model was optimized from 25 to 400 °C and frequencies from 20 to 100 MHz [33]. Table 1 is a list of the measured circuit parameters used as inputs in the model.

Table 2 is a list of the optimized parameters R_g_, R_s_, R_d_, L_g_, L_s_, L_d_, R1, and R2 at temperatures from 25 to 400 °C that were used to match the simulated S-parameters with the measured parameters. As expected, the capacitive and inductive components listed in Table 1 and Table 2 exhibited a small relative increase due to thermionic losses while the resistive elements showed a substantial increase. The transconductance, g_m_, decreased over this temperature range due to the significantly large relative increases in the internal resistances R1 and R2.

**Figure 9:**
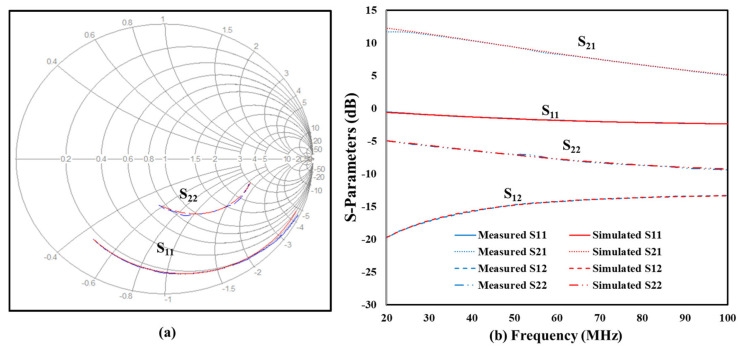
Measured (blue) and simulated (red) S-parameters of the SiC SIT at 400 °C and a frequency range from 20 MHz to 100 MHz: (**a**) Smith chart of S_11_ and S_22_, and (**b**) S-parameters.

The accuracy of the SiC SIT model was determined by assessing the difference between the measured and modeled S-parameters between 25 and 400 °C with an emphasis at 400 °C and 50 MHz. The high-frequency simulated and measured S-parameters at 400 °C are shown in Figure 9. Figure 9a is a Smith chart representation of the input and output reflection coefficients between 20 and 100 MHz. Both the measured and simulated responses for S_11_ and S_22_ were in good agreement. Figure 9b shows the simulated and measured values S_11_, S_21_, S_12_, and S_22_ between 20 and 100 MHz. The differences between the measured and simulated data for S_11_, S_21_, S_12_, and S_22_ were virtually indistinguishable, indicating strong agreement between the model and device performance. At 400 °C, the unmatched transistor gain, S_21_, was above 0 dB and the isolation, S_12_, was below −13 dB over the entire frequency range, which are promising features for a transistor operating simultaneously at high frequencies and high temperatures. S_11_ and S_22_ indicate that the transistor was not matched to the input and output 50 Ω ports, which was expected because a matching network was not incorporated in the measurements or simulations. A comparison of the measured and simulated data indicates that the SiC SIT small signal model accurately reflects the device performance for frequencies between 20 and 100 MHz and temperatures up to 400 °C.

To evaluate the accuracy of the SiC SIT model at high temperatures, the transition (cut-off) frequencies, *f_t_*, for temperatures up to 400 °C, were calculated using simulated S-parameters from the model and compared to the transition frequencies calculated using the measured S-parameters as well as the data presented in Figure 7. The transition frequency was defined as the frequency at which the current gain, H_21_, reaches unity (or H_21_ dB = 0). H_21_ was determined from the measured and simulated S-parameters using Equation (3):

(3) $$H_{21}=\frac{{-2S}_{12}}{\left(1-S_{11}\right)\left(1-S_{22}\right)+S_{12}S_{21}}.$$

**Figure 10:**
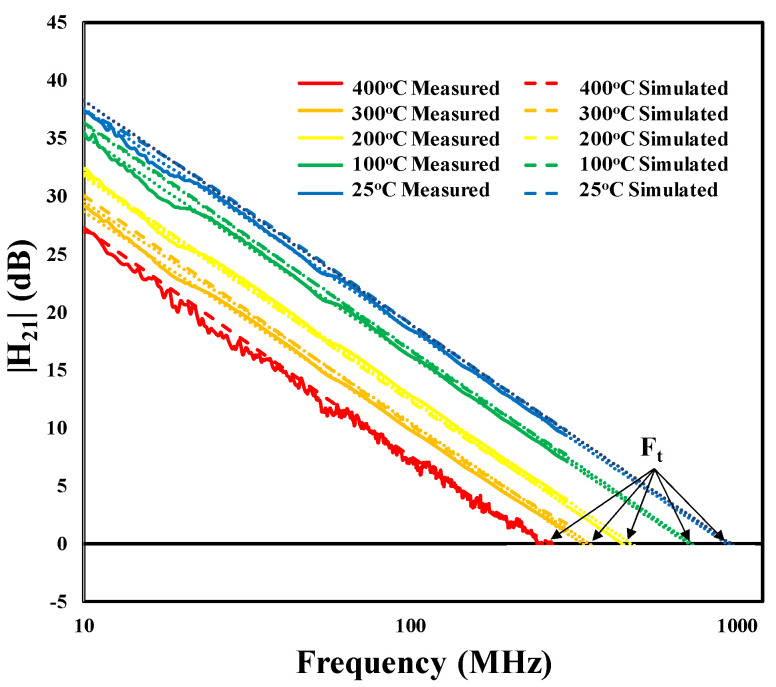
Plots of measured and simulated current gain (H_21_) as a function of frequency for temperatures between 25 and 400 °C. The dotted lines represent a linear extrapolation of the measurement data to determine f_t_ for frequencies that exceeded the measurement range (300 MHz).

**Figure 11:**
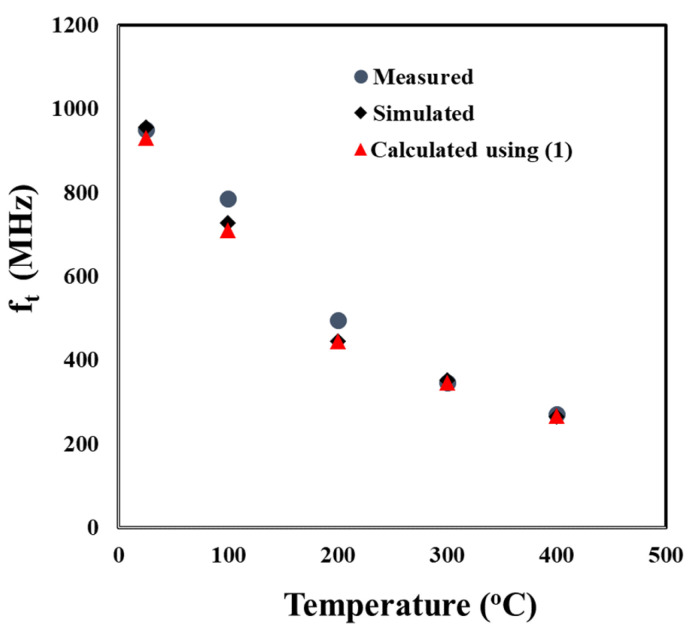
Calculated, simulated, and measured values of the transition frequency, f_t_, for temperatures between 25 and 400 °C.

Figure 10 presents the current gain versus frequency for the measured and simulated S-parameters up to 400 °C. For frequencies exceeding 300 MHz, the transition frequency was determined by extrapolating a best-fit line of the measured data to the horizontal axis. Figure 11 shows that the *f_t_* from the simulated S-parameters closely matched the *f_t_* from the measured S-parameters over the entire measured frequency range, the difference being only 0.8% at 400 °C.

Figure 11 compares the *f_t_* calculated from the simulated S-parameters, those calculated from the measured S-parameters, and those calculated from the transconductance measurements (Figure 7) for temperatures up to 400 °C. At 400 °C, the greatest difference was between the *f_t_* calculated from the simulated S-parameters and the measured S-parameters at only 1.8%. The smallest difference at 400 °C was between the *f_t_* calculated from the simulated S-parameters and the measured transconductance values at 0.3%. These small differences indicate that the small signal model accurately reflects the behavior of the SiC SIT.

The small signal model was further evaluated using model outputs to calculate the maximum frequency where the unilateral power gain, *U*, becomes unity, *f_max_*, and comparing this frequency to calculations made using the experimentally measured values. The unilateral power gain, *U*, is determined using Equation (4):

(4) $$U=\frac{{\left|\frac{S_{21}}{S_{12}}-1\right|}^{2}}{2*K*\left(\left|\frac{S_{21}}{S_{12}}\right|\right)-2*Re(\frac{S_{21}}{S_{12}})}$$

where *K* is defined as the stability factor and can be found using:

(5) $$K=\frac{1+{∆}^{2}-{\left|S_{11}\right|}^{2}-{\left|S_{22}\right|}^{2}}{2*|S_{12}S_{21}|}$$

where Δ is the determinant of the two-port S-parameter matrix, which can be found using:

(6) $$\Delta =\left|S_{11}S_{22}-S_{12}S_{21}\right|.$$

**Figure 12:**
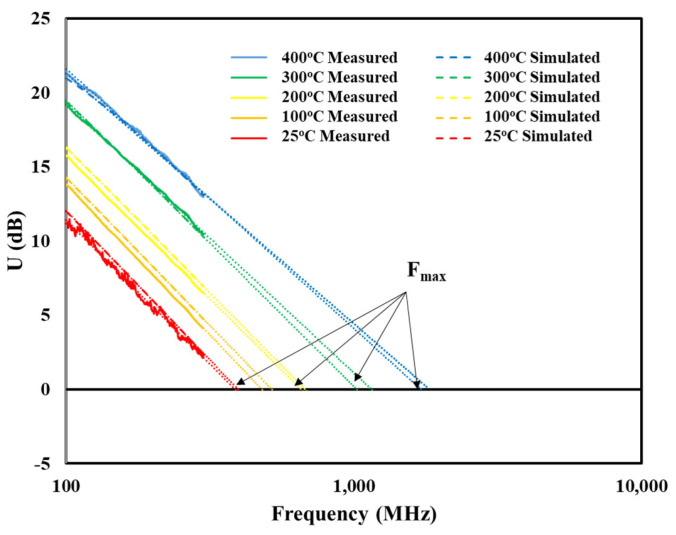
Plots of unilateral power gain (U) versus frequency for temperatures between 25 °C and 400 °C from the measured and simulated data. Linear extrapolation of the data above 300 MHz was performed to determine f_max_.

**Figure 13:**
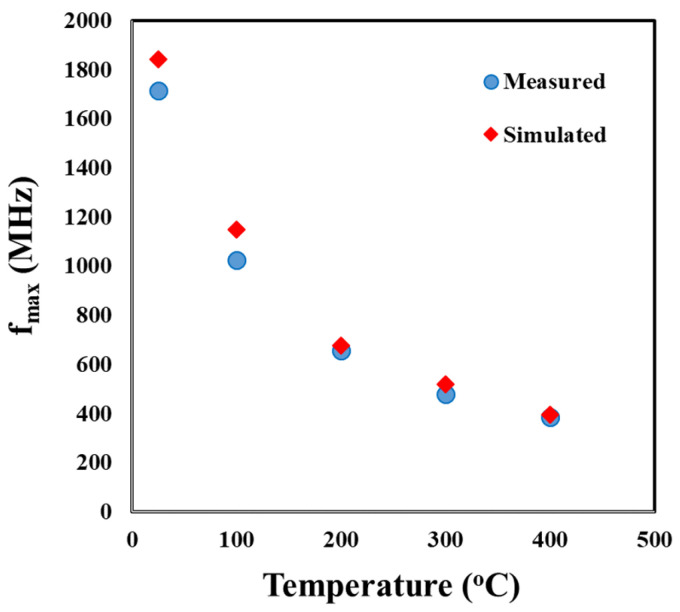
Measured and simulated f*_max_* for temperatures between 25 and 400 °C.

Figure 12 shows the unilateral power gain calculated from simulated and measured S-parameters versus frequency for temperatures up to 400 °C. Like the transition frequency, *f_max_* was defined as the frequency at which the unilateral power gain reaches unity (or *U* dB = 0). As was carried out for the transition frequency, *f_max_* values greater than 300 MHz were determined by extrapolating a best-fit line of the measured data to the horizontal axis. Figure 13 compares the *f_max_* versus temperature for *f_max_* values calculated using both the simulated and measured S-parameters. At 400 °C, the *f_max_* from the measured S-parameters was 396 MHz while that from the simulated was 386 MHz, a difference of only 2.5%, indicating strong agreement between the SIT SiC model and measured device characteristics. This analysis further supports the accuracy of the small signal model and provides additional evidence that the SiC SIT is a viable candidate to be used in amplifier, oscillator, and other active circuitry for RF applications operating at 400 °C.

**Figure 14:**
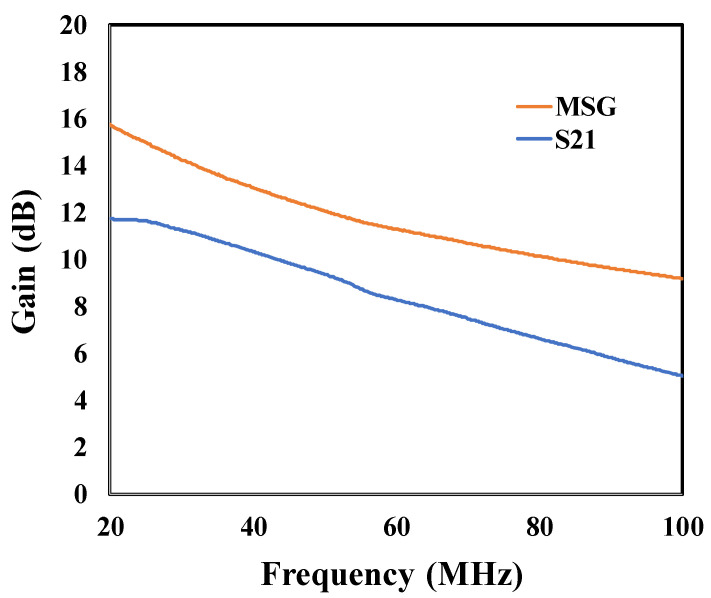
Plots of maximum stable gain (MSG) and S_21_ for the SiC SIT at 400 °C.

Figure 14 presents the maximum stable gain (MSG) as a function of frequency at 400 °C for the SIT. Defined as the ratio of magnitude of S_21_ to the magnitude of S_12_ when the stability factor is less than unity, the MSG defines a maximum practical limit to the gain. Figure 14 shows that at 400 °C, the MSG for the SiC SIT ranged from 16 dB to 10 dB between 20 MHz and 100 MHz and was 12 dB at 50 MHz. These findings suggest that the SiC SIT is suitable for use in a wide range of high-temperature sensor interfaces that incorporate amplifier circuits with operating frequencies between the 20 and 100 MHz range and a gain of nominally 10 dB.

## 4. Conclusions

This paper describes the high-temperature characterization of a commercially available silicon carbide static induction transistor (SIT) and the subsequent development of a small signal model that emulated its operation from 25 to 400 °C and frequencies between 20 and 100 MHz. For an I_ds_ of 40 mA, measurements performed at 400 °C revealed the following for this transistor:

- A S_21_ gain of 9.35 dB, which was only a ~40% reduction compared to its room temperature value.
- A transconductance, g_m_, of 60 mS, which corresponded to a transition frequency, *f_t_*, of 270 MHz. Moreover, the transconductance decreased by only a factor of ~3 between 25 and 400 °C.

These findings indicate that when operated under low power conditions, this high power SiC SIT exhibits sufficient gain for use in RF circuits designed to operate at temperatures up to 400 °C and frequencies in the 50 MHz range.

Utilizing both DC and high frequency measurements as input parameters, a small signal model was developed for this transistor using the circuit simulator, ADS. Comparing the modeling results to the measurement values revealed the following for operation at 400 °C:

- S-parameters generated from the small signal model differed from the measured values by less than 2%.
- The transition frequency, *f_t_* calculated from the small signal model differed from *f_t_* calculated using the measured S-parameters by less than 2%.
- The maximum frequency, *f_max_*, calculated using S-parameters from the small signal model differed from that calculated from measurement data by only 2.5%.
- The maximum stable gain was 12 dB at 50 MHz and ranged from 16 dB at 20 MHz to 10 dB at 100 MHz.

These comparisons indicate that this model accurately represents the RF performance of the SiC SIT for temperatures up to 400 °C and predicts that the transistor will have sufficient gain for sensor interface circuits operating in this temperature range.

To the best of our knowledge, this is the first time a SiC SIT has been characterized up to 400 °C under both DC and high frequency conditions, and the first time a model for the SiC SIT has been developed for high-temperature and high-frequency operation. This work should stimulate further development of the SiC SIT for wireless RF devices operating at high temperature, in particular, sensor systems that require the amplification of signals at the measurement location to include reliability, aging, and long-term stress testing.

## Figures and Tables

**Table 1:** Measured circuit parameters used as inputs in the SiC SIT model.

Temp (°C)	25	100	200	300	400	% Difference
C_gs_ (pF)	22.0	22.0	22.5	23.1	23.4	6.1
C_ds_ (pF)	0.51	0.53	0.54	0.57	0.58	12.0
C_gd_ (pF)	11.5	11.4	11.5	11.8	12.2	6.08
g_m_ (mS)	200	165	115	80	60	−70

**Table 2:** Simulated circuit parameters determined from the optimized SiC SIT model.

Temp (°C)	25	100	200	300	400	% Difference
R_g_ (Ω)	0.95	1.24	1.5	1.77	2.13	124
R_s_ (Ω)	0.12	0.15	0.24	0.38	0.50	303
R_d_ (Ω)	1.12	2.32	3.56	4.68	5.84	421
L_g_ (nH)	1.48	1.48	1.48	1.51	1.51	2.02
L_s_ (nH)	1.16	1.16	1.16	1.16	1.16	0.00
L_d_ (nH)	1.48	1.48	1.51	1.51	1.51	2.02
R1 (kΩ)	0.68	1.87	2.31	3.80	4.21	512
R2 (Ω)	52.2	75.3	125	168	200	283

## Data Availability

The datasets presented in this article are not readily available because the data are part of an ongoing study but are also owned and maintained by the U.S. Government. Requests to access the datasets should be directed to Maximilian Scardelletti (maximilian.c.scardelletti@nasa.gov).
